# Perception Disparity of Telemedicine Use between Outpatients and Medical Staff during the COVID-19 Pandemic

**DOI:** 10.3390/healthcare10101965

**Published:** 2022-10-08

**Authors:** Jia-Jyun Wu, Chieh-Liang Wu, Meng-Hsun Lee, Chieh-Chung Huang, Yi-Jhen Huang, Pi-Shan Hsu

**Affiliations:** 1 Department of Family Medicine, Taichung Veterans General Hospital, Taichung 40705, Taiwan; 2 Department of Post-Baccalaureate Medicine, College of Medicine, National Chung Hsing University, Taichung 40227, Taiwan; 3 Department of Critical Care Medicine, Taichung Veterans General Hospital, Taichung 40705, Taiwan; 4 Department of Medical Administration, Taichung Veterans General Hospital, Taichung 40705, Taiwan; 5 Computer and Communications Center, Taichung Veterans General Hospital, Taichung 40705, Taiwan

**Keywords:** COVID-19, telemedicine, telehealth, telephone visit, perception, outpatients

## Abstract

We assessed the characteristics and perception of telephone appointments among outpatients and medical staff during the COVID-19 pandemic in Taiwan. Our survey was performed by giving self-administered questionnaires to the enrollees. Basic socioeconomic status data were collected. We used a valid and reliable telehealth usability questionnaire (TUQ) to assess the telemedicine experience among outpatients and medical staff. Only outpatients with chronic illness and who had regular visits before the pandemic were enrolled. We delivered the questionnaire survey to participants who used telephone appointments from 20 May 2021 to 31 July 2021 in Taichung Veterans General Hospital. A total of 471 outpatients and 203 medical staff completed the survey. Most of the respondents were aged 30–69, college-educated, women, and married. Outpatients have higher scores in all dimensions of TUQ than medical staff, especially in the dimensions of ease of use and effectiveness. Age, gender, education, and marriage have no significant associations in the medical staff group. In the outpatient group, gender is the only significant factor in the six dimensions of TUQ. We found a significant disparity in the perception gap of telemedicine among outpatient and medical staff. Outpatients are satisfied with telephone appointments during the COVID-19 pandemic, but medical staff are concerned about the ease of use and effectiveness.

## 1. Introduction

Since the outbreak of the novel coronavirus disease 2019 (COVID-19) in early 2020, the evolution of telemedicine has grown drastically worldwide. Healthcare systems are dedicated to developing telemedicine in many countries, including future applications, disease prevention and control, medical quality, and economics. A recent study reported that telemedicine should not only be a temporary measure in times of an emergency pandemic; it evidently has the potential to care for outpatients during the pandemic and beyond [1].

Taiwan is one of the fastest aging societies in the world and is set to become super-aged by 2025 [2]. With increased long-term care needs and the development of information and communications technology, the Ministry of Health and Welfare in Taiwan commenced “Regulations of Treatment on Telemedicine” on 11 May 2018 to improve accessibility. However, the regulations only applied to those who lived in the mountains, outlying islands, remote areas, or other particular conditions [3]. Due to the government’s effective response to COVID-19, Taiwan’s medical system did not have great difficulty promoting telemedicine in 2020. Nevertheless, as COVID-19 started to surge in mid-May 2021, the government in Taiwan loosened the regulations. Outpatients with chronic diseases could use telemedicine, such as video or telephone conferences, for routine checks. The telemedicine regulations were relaxed until one month after the Level 3 epidemic alert lifted. Telemedicine provided continuity of care during the COVID-19 pandemic and was demonstrated as an effective solution for chronic illness management [4].

The use of telephone appointments increased significantly more than video appointments in Taichung Veterans General Hospital. Besides, the annual survey of the National Health Insurance (NHI) in Taiwan showed a satisfaction disparity between physicians and patients. Several studies have shown positive attitudes and satisfaction toward telemedicine among clinicians and outpatients [4,5,6,7]. However, there is a lack of research regarding user experience and perception gaps of telephone appointments among patients and physicians, especially in the outpatient clinic setting. For the further development of telemedicine, it is necessary to analyze the user experiences and perception gaps of telephone appointments in the outpatient clinic during the COVID-19 pandemic.

## 2. Materials and Methods

Our survey took the form of a self-administered questionnaire. We collected data on age, gender, basic socioeconomic profiles, and questions about telemedicine experience. Our project had two parts; one for the outpatient group and one for the medical staff group. The target population for the study was patients who had telephone appointments in Taichung Veterans General Hospital from 20 May 2021 to 31 July 2021. In total, 204 physicians, 183 nurses, and 1790 outpatients participated in the telephone appointments.

To our knowledge, there were few existing validated questionnaires in Asia to evaluate the user characteristics and experiences of telephone appointments in the outpatient clinic. We designed a questionnaire, which is an anonymous survey. It had two versions; one for outpatients and one for medical staff. The questionnaire includes gender, age, education, marriage, identity (outpatient, family member, or medical staff), and user experience. The telehealth usability questionnaire (TUQ) was used widely in surveys of telehealth [8,9]. We revised the TUQ to a six-dimension survey, including usefulness, ease of use, effectiveness, reliability, willingness to re-visit, and satisfaction (Table 1). Each dimension of the questions contains five scores, 1 for strongly disagree, 2 for disagree, 3 for no opinion, 4 for agree, and 5 for strongly agree. We gathered a focus group that enrolled six experts to assess the content validity. The mean item-level content validity indices of outpatient and medical staff versions were 0.97 and 1.00, respectively. The scale-level content validity indices, based on the universal agreement calculation method of outpatient and medical staff versions, were 0.83 and 1.00, respectively. We also evaluated the internal consistency. The Cronbach’s alpha was 0.955.

We designed a purposive sampling by sending text messaging questionnaires to people who had telephone appointments from 20 May 2021 to 31 July 2021 in Taichung Veterans General Hospital, aided by information technology technicians. The outpatients and their family members who assisted with the telephone appointment or the associated medical staff were enrolled. An agreement to participate in the questionnaire survey was required. We excluded people on the first appointment, those who did not agree to the questionnaire survey, and those aged 20 and younger. The study was approved by the ethical review committee conducted by the Institutional Review Board Taichung Veterans General Hospital (CE21288A).

Descriptive statistics were analyzed to obtain characteristics and summarize participants’ telephone appointment experiences during the COVID-19 pandemic. A Chi-Square test was used to examine the relationships between questionnaire dimensions from two sample groups. We also use multiple regression of variables within both groups to examine the association. All analyses were performed using SPSS for Windows version 16.0 (SPSS Institute Inc., Chicago, IL, USA).

## 3. Results

In total, 471 outpatients and 203 medical staff responded to the questionnaire. The response rates were 26.31% in the outpatient group and 52.45% in the medical staff group. The predominant age group of outpatients and medical staff was 30–69 years. Most of the respondents were women and married. The education level of both groups is mainly at the college level. Most (78.56%) of respondents to the outpatient survey were filled in by patients themselves (Table 2).

**Figure 1:**
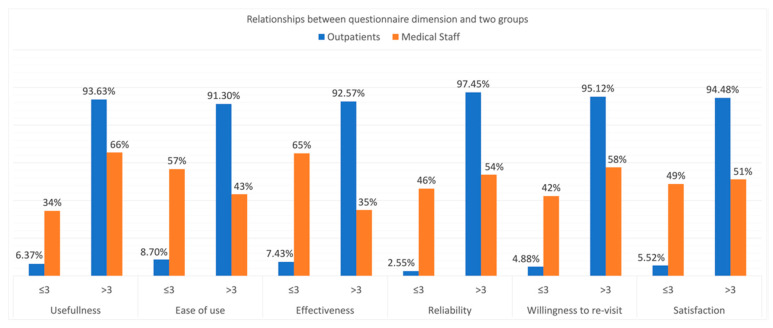
Relationships between the questionnaire dimension and two groups.

The question statements in the TUQ were slightly different in the two versions for outpatients and medical staff, but the main six dimensions were the same. In the outpatient group, respondents gave scores higher than four in all six dimensions. However, the scores were lower in the medical staff group, especially in dimensions 2 (ease of use) and 3 (effectiveness) (Table 3). The difference is significant between the two groups (Figure 1).

There is no significant difference in the analysis of variances in each dimension in the medical staff group, except for the respondent identity in dimension 3 (effectiveness). In the outpatient group, gender is a significant variable in the six dimensions (Table 4). In the multiple regression, gender is still a significant variable in the six dimensions among the outpatient group. We also used the variance-inflation factors for detecting multicollinearity.

## 4. Discussion

In our research, we found an evident gap in the perception of telemedicine. Outpatients gave a higher score than medical staff in all six dimensions of TUQ. In the outpatient group, patients themselves are less willing to re-visit than family and friends, which has a significant relationship. In the medical staff group, nurses gave a higher score in the effectiveness dimension than physicians. Women were more satisfied with telephones than men in the outpatient group. In the multiple regression model, age, education, and marriage have no significant associations among the six dimensions in our questionnaire.

The overall satisfaction results are compatible with the annual survey of the NHI in Taiwan. In the latest NHI satisfaction survey in 2019, people had high satisfaction with the NHI service at 89.7%, while medical staff only had 33.7% [10]. In our telephone appointments survey, the percentage of scores higher than three in the six dimensions is over 90% in the outpatient group. A Taiwan government survey in 2011 found the average time of in-person outpatient appointments is 10.2 min, which is also consistent with the time of telephone appointments.

A recent study about video telemedicine using the TUQ in the COVID-19 pandemic has shown that the average usability score (scale 1–5) was 3.87, with the highest dimensions in the usefulness (μ = 4.29) and physician satisfaction (μ = 4.13) and the lowest dimension in reliability (μ = 3.02) [11]. Our study demonstrated similar results; the average usability score was 3.42, with the highest in the usefulness dimension and the lowest in the effectiveness dimension. The disparity might be related to several factors. The medical staff had little experience with telemedicine before the pandemic. Research has shown most physicians did not get training in telemedicine [11,12]. It is urgent for medical staff to adopt the new model of outpatient appointments. A telemedicine study also revealed communication is an essential concern of the medical staff [13]. Physicians have more confidence in traditional in-person appointments because of physical exams, eye contact, and body language. Besides, telemedicine may also increase the burden on physicians.

A systemic review of patient experience with telemedicine has demonstrated that the most significant benefits were the time saved, better accessibility, convenience, and cost-efficiency. Age did not have significant associations among the satisfaction levels. Challenges with technical issues and lack of physical examination were the main concern encountered in telemedicine [14]. These findings are compatible with our results. In the view of outpatients in Taiwan, accessibility and convenience were important considerations, especially during the COVID-19 pandemic. However, the medical staff were more concerned about the safety and effectiveness of the new technology than the convenience. An interview study of physicians’ perception of telehealth also revealed doubts and uncertainty about telemedicine, such as efficiency, safety, and the adequacy of current regulations [15].

Our study has several strengths. It is the first study to evaluate telephone usability using TUQ among medical staff and outpatients. We revised the TUQ to a six-dimension survey with confirmed reliability and validity. We found a significant perception disparity of telemedicine among outpatients and medical staff. Outpatients with chronic illnesses were satisfied with the telephone appointment for regular prescriptions during the pandemic. However, medical staff considered patient safety and effectiveness more and felt unsatisfied with the current use of telephone appointments. The policymakers and administration need to enhance the telemedicine tools and programs to reduce the burden and increase patient safety in future developments.

There are several limitations to our study. First, it was a cross-sectional anonymous internet questionnaire survey with a limited number of respondents. There was a low response rate in the outpatient group, as those nonrespondents might be less educated or interested in our research topic. Because it was an anonymous self-administered questionnaire survey, we could not follow up to improve the response rate and compare the difference between respondents and nonrespondents. Second, we did not collect some socioeconomic data, such as income or urbanicity of residence in the outpatient group. Personal medical history and adherence were also not controlled. Third, only people who had outpatient appointments before the pandemic were enrolled. We exclusively evaluated audio telehealth without video telehealth. The study is not generalized to other populations. Further studies are required to explore telemedicine usability. There is still little known about characteristics among subgroups in the medical staff or time using telemedicine.

## 5. Conclusions

We found a significant disparity in the perception gap of telemedicine among outpatients and medical staff. Outpatients were satisfied with telephone appointments during the COVID-19 pandemic, but medical staff were concerned about the ease of use and effectiveness. Further studies are required to explore the characteristics among subgroups.

## Figures and Tables

**Table 1:** The telehealth usability questionnaire (TUQ) of TCVGH ^1^.

Dimensions	Questions
1. Usefulness	Is it easy to get the telephone appointment information and an appointment?
2. Ease of use	Is the telephone system simple and easy to use?
3. Effectiveness	Did the telephone appointment provide information in the same manner as the previous in-person appointment?
4. Reliability	Do you think a telephone appointment has the same reliability as an in-person appointment?
5. Willingness to re-visit	Would you use a telephone appointment again?
6. Satisfaction	Are you satisfied with the telephone system?

^1^ TCVGH: Taichung Veteran General Hospital.

**Table 2:** Sociodemographic data of the participants who received telephone appointments during the COVID-19 pandemic in Taiwan.

Variables	Outpatients (*n* = 471)	Medical Staff (*n* = 203)
Age group (years), *n* (%)		
≤19	17 (3.61)	-
20–29	17 (3.61)	37 (18.23)
30–39	49 (10.40)	77 (37.93)
40–49	95 (20.17)	45 (22.17)
50–59	94 (19.96)	39 (19.21)
60–69	111 (23.57)	4 (1.97)
70–79	46 (9.77)	1 (0.49)
≥80	42 (8.91)	-
Gender, *n* (%)		
Men	186 (39.49)	91 (44.83)
Women	285 (60.51)	112 (55.17)
Education, *n* (%)		
Illiteracy	18 (3.82)	-
Elementary school	33 (7.01)	-
Junior high school	26 (5.52)	-
Senior high school	103 (21.87)	-
College	213 (45.22)	140 (68.97)
Graduate school	78 (16.56)	63 (31.03)
Marriage, *n* (%)		
Married	441 (93.63)	136 (67.00)
Unmarried	25 (5.31)	67 (33.00)
Other	5 (1.06)	-
Respondents, *n* (%)		
Patient themselves	370 (78.56)	
Family or friends	101 (21.44)	
Respondents, *n* (%)		
Physicians		131 (64.53)
Nurses		72 (35.47)

**Table 3:** Questionnaire dimensions of the participants who received telephone appointments during the COVID-19 pandemic in Taiwan.

Questionnaire Dimension	Scales, *n* (%)	Outpatients (*n* = 471)	Medical Staff (*n* = 203)
1. Usefulness: Is it easy to get the telephone appointment information and an appointment?	1-strongly disagree	2 (0.42)	8 (3.94)
	2-disagree	5 (1.06)	14 (6.90)
	3-no opinion	23 (4.88)	48 (23.65)
	4-agree	144 (30.57)	85 (41.87)
	5-strongly agree	297 (63.06)	48 (23.65)
2. Ease of use: Is the telephone system simple and easy to use?	1-strongly disagree	6 (1.27)	18 (8.87)
	2-disagree	9 (1.91)	30 (14.78)
	3-no opinion	26 (5.52)	67 (33.00)
	4-agree	135 (28.66)	59 (29.06)
	5-strongly agree	295 (62.63)	29 (14.29)
3. Effectiveness: Did the telephone appointment provide information in the same manner as the previous in-person appointment?	1-strongly disagree	1 (0.21)	10 (4.93)
	2-disagree	7 (1.49)	49 (24.14)
	3-no opinion	27(5.73)	73 (35.96)
	4-agree	163 (34.61)	55 (27.09)
	5-strongly agree	273 (57.96)	16 (7.88)
4. Reliability: Do you think a telephone appointment has the same reliability as an in-person appointment?	1-strongly disagree	1 (0.21)	9 (4.43)
	2-disagree	2 (0.42)	23 (11.33)
	3-no opinion	9 (1.91)	62 (30.54)
	4-agree	143 (30.36)	85 (41.87)
	5-strongly agree	316 (67.09)	24 (11.82)
5. Willingness to re-visit: Would you use a telephone appointment again?	1-strongly disagree	4 (0.85)	13 (6.40)
	2-disagree	5 (1.06)	24 (11.82)
	3-no opinion	14 (2.97)	49 (24.14)
	4-agree	95 (20.17)	75 (36.95)
	5-strongly agree	353 (74.95)	42 (20.69)
6. Satisfaction: Are you satisfied with the telephone system?	1-strongly disagree	6 (1.27)	9 (4.43)
	2-disagree	2 (0.42)	22 (10.84)
	3-no opinion	18 (3.82)	68 (33.50)
	4-agree	124 (26.33)	76 (37.44)
	5-strongly agree	321 (68.15)	28 (13.79)

**Table 4:** Analysis of variance for respondents’ identity, gender, age, education, and marriage.

Dimension	Outpatients *p* Value	Medical Staff *p* Value
**Dimension 1**		
Respondents’ identity	0.842	0.213
Gender	<0.0005	-
Age	0.386	0.520
Education	0.120	-
Marriage	0.436	0.051
**Dimension 2**		
Respondents’ identity	0.468	0.157
Gender	0.017	-
Age	0.664	0.862
Education	0.731	-
Marriage	0.655	0.163
**Dimension 3**		
Respondents’ identity	0.841	0.010
Gender	0.002	-
Age	0.652	0.916
Education	0.533	-
Marriage	0.250	0.110
**Dimension 4**		
Respondents’ identity	0.797	0.143
Gender	0.019	-
Age	0.594	0.789
Education	0.785	-
Marriage	0.247	0.614
**Dimension 5**		
Respondents’ identity	0.004	0.290
Gender	0.007	-
Age	0.470	0.070
Education	0.677	-
Marriage	0.661	0.159
**Dimension 6**		
Respondents’ identity	0.127	0.226
Gender	0.003	-
Age	0.179	0.584
Education	0.605	-
Marriage	0.762	0.732

## Data Availability

The datasets used and analyzed during the current study are available from the corresponding authors on reasonable request.
